# Medical Fees

**Published:** 1845-12

**Authors:** 


*Medical Fees.*—Persons generally are not aware of the importance of a fee. A physician can make a more correct diagnosis, and his remedies will operate much more beneficially where he is certain of being paid, than when he proceeds without reference to "the recompense of reward." A fee inspires the practitioner with confidence in himself and faith in his remedies, which is almost as important as the exercise of faith on the part of the patient. An eminent English physician once remarked that he never felt his own pulse, nor looked at his tongue in the glass, without unconsciously slipping a guinea from one pocket into the other. Let those, therefore, who wish to derive the full benefit of a physician's advice, bear in mind the necessity of a fee.—*St. Louis Med. & Surg. Jour.*

